# Variation in photosynthetic induction between rice accessions and its potential for improving productivity

**DOI:** 10.1111/nph.16454

**Published:** 2020-03-03

**Authors:** Liana G. Acevedo‐Siaca, Robert Coe, Yu Wang, Johannes Kromdijk, W. Paul Quick, Stephen P. Long

**Affiliations:** ^1^ Department of Crop Sciences University of Illinois at Urbana‐Champaign Urbana IL 61801 USA; ^2^ C_4_ Rice Center International Rice Research Institute Los Baños Laguna 4031 Philippines; ^3^ High Resolution Plant Phenomics Centre Commonwealth Scientific and Industrial Research Organization (CSIRO) Plant Industry Canberra ACT 2601 Australia; ^4^ Carl R. Woese Institute for Genomic Biology University of Illinois at Urbana–Champaign Urbana IL 61801 USA; ^5^ Department of Plant Sciences University of Cambridge Cambridge CB2 3EA UK; ^6^ Department of Animal and Plant Sciences University of Sheffield Western Bank Sheffield S10 2TN UK; ^7^ Department of Plant Biology University of Illinois at Urbana–Champaign Urbana IL 61801 USA; ^8^ Lancaster Environment Centre Lancaster University Lancaster LA1 4YQ UK

**Keywords:** dynamic photosynthesis, food security, photosynthesis, photosynthetic induction, rice, Rubisco activase, stomata, water‐use efficiency

## Abstract

- Photosynthetic induction describes the transient increase in leaf CO_2_ uptake with an increase in light. During induction, efficiency is lower than at steady state. Under field conditions of fluctuating light, this lower efficiency during induction may cost > 20% of potential crop assimilation. Accelerating induction would boost photosynthetic and resource‐use efficiencies.
- Variation between rice accessions and potential for accelerating induction was analysed by gas exchange. Induction during shade to sun transitions of 14 accessions representing five subpopulations from the 3000 Rice Genome Project Panel (3K RGP) was analysed.
- Differences of 109% occurred in the CO_2_ fixed during the first 300 s of induction, 117% in the half‐time to completion of induction, and 65% in intrinsic water‐use efficiency during induction, between the highest and lowest performing accessions. Induction in three accessions with contrasting responses (AUS 278, NCS 771 A and IR64‐21) was compared for a range of [CO_2_] to analyse limitations. This showed *in vivo* capacity for carboxylation at Rubisco (*V*_c,max_), and not stomata, as the primary limitation to induction, with significant differences between accessions.
- Variation in nonsteady‐state efficiency greatly exceeded that at steady state, suggesting a new and more promising opportunity for selection of greater crop photosynthetic efficiency in this key food crop.

## Abstract 2

See also the Commentary on this article by McAusland & Murchie, **227**: 989–991.

## Introduction

The efficiency of light interception and conversion to biomass through photosynthesis strongly affects the yield potential of a crop cultivar (Monteith, 1978; Zhu *et al.*, 2010). Understanding of limitations to crop photosynthesis for yield increases have focused on rates of leaf CO_2_ uptake under conditions of constant light‐saturation. However, under field conditions, leaves within a crop canopy experience continually changing light intensity due to intermittent cloud cover, movement of leaves in the wind, and changing solar angles (Walker, 1972; Pearcy, 1990; Burgess *et al.*, 2016). As a result, the photon flux density at any point in the canopy is in continual change, with order of magnitude changes occurring in a matter of seconds (Pearcy, 1990; Pearcy *et al.*, 1996; Zhu *et al.*, 2004; Slattery *et al.*, 2018; De Souza *et al.*, 2019). Dynamic measurements provide insight as to how a plant reacts to the rapid changes in light that occur in crop canopies and can be used to identify limitations to photosynthesis that might be improved. Transient photosynthetic responses can be categorized by the direction of the light intensity change; that is, from low light to high light or vice versa. This study focuses on the former, photosynthetic induction on shade‐to‐sun transitions. Induction is considered to be affected by four main processes: photoactivation of enzymes involved in the regeneration and production of ribulose 1,5‐bisphosphate (RuBP), build‐up of concentrations of the intermediates of carbon (C) metabolism, the activation of Rubisco, and the opening of stomata (Pearcy *et al*, 1994; Mott & Woodrow, 2000; Slattery *et al.*, 2018; Deans *et al.*, 2019). By definition, photosynthetic efficiency during induction is lower than at steady state, representing a loss of potential crop CO_2_ uptake (Pearcy *et al.*, 1994; Mott & Woodrow, 2000; Deans *et al.*, 2019). In the case of wheat, this loss over the course of a day was calculated at 21% (Taylor & Long, 2017). If induction could be accelerated, these losses could be reduced and intrinsic water‐use efficiency increased (Carmo‐Silva *et al.*, 2014; Lawson & Blatt, 2014; McAusland *et al.*, 2016; Vialet‐Chabrand *et al.*, 2017).

Natural genetic variation within crop germplasm forms the basic breeding material to develop new cultivars, and its utilization could aid in improving photosynthetic efficiency, including induction (Flood *et al.*, 2011; Lawson *et al.*, 2012; Driever *et al.*, 2014; Gu *et al.*, 2014; De Souza *et al.*, 2019). Previous experiments have studied photosynthetic induction in soybean and cassava, identifying considerable variation between genotypes (Sakoda *et al.*, 2016; Soleh *et al.*, 2016, 2017; De Souza *et al.*, 2019).

Rice is a direct source of calories for more people than any other single crop. It also serves as the main staple for some 560 million chronically hungry people on the Asian continent (Mohanty, 2013). Improving its photosynthetic efficiency has become a focus, in particular by introducing C_4_ photosynthesis (Kajala *et al.*, 2011). Breeding for increased speeds of induction might provide a more immediate and complementary means to increase photosynthetic efficiency (Wang *et al.*, 2020).

Here, the photosynthetic induction of 14 accessions from the 3000 Rice Genome Project (3K RGP) was quantified. These were selected to represent five subpopulations and seven diverse geographical regions. Rubisco activase (Rca) is an important mediator of photosynthetic induction through activation of Rubisco. In rice, it is coded by a single gene that is alternatively spliced to give alpha and beta‐isoforms (To *et al.*, 1999). Given the importance of Rca, accessions were also selected on mismatches in the genetic sequence for Rca. The objectives were, first, to compare, quantify and characterize the photosynthetic induction in rice relative to steady state and, second, to examine the response of photosynthetic induction in rice at different [CO_2_] to deduce *in vivo* limitations to induction.

## Materials and Methods

### Germplasm and growing conditions

Fourteen accessions representing five rice subpopulations (*indica*, tropical *japonica*, temperate *japonica*, aus, and aromatic), seven geographical regions, and different canopy structures were taken from the 3K RGP held at the International Rice Research Institute (IRRI) in Los Baños, Philippines (Supporting Information Fig. S1; Table 1). Further selection from single nucleotide polymorphisms (SNPs) was made using the International Rice Informatics Consortium Rice SNP‐Seek Database managed by IRRI (Table S2).

**Table 1:** Minimum, maximum, mean, percentage genetic variation (PGV), and significance of variation assessed by ANOVA for each steady‐state and non‐steady‐state measure across 14 rice (*Oryza sativa*) accessions.

Trait	Unit	Min.	Max.	Mean	PGV (%)	df	*P*‐value	*P*
**Dynamic traits**								
${\bar{A}}_{300}$	µmol m^−2^ s^−1^	5.8	18	12	101.6	13	<0.0001	***
${\bar{A}}_{700}$	µmol m^−2^ s^−1^	9.79	23.81	19.75	71	13	<0.0001	***
*g*_s avg_	mol m^−2^ s^−1^	0.1	0.71	0.33	189.6	13	<0.0001	***
iWUE_avg_	µmol CO_2_ mol^−1^ H_2_O	22.6	71.01	40.13	120.9	13	0.026	*
$C_{i\text{avg}}$	µmol mol^−1^	260.3	341.3	310.3	26.1	13	0.07	
*A*_300_	µmol m^−2^ s^−1^	10.3	24	16.8	81.7	13	<0.0001	***
*A*_Max_	µmol m^−2^ s^−1^	11.8	32.9	21.5	98.1	13	<0.0001	***
IT_90_	s	27.3	127.5	65.9	152	13	0.0014	**
IT_90_	s	99	289.6	201.9	94	13	0.216	
IT_90_ − IT_50_	s	54.18	229.6	136	129	13	0.519	
C Loss_300_	µmol m^−2^ s^−1^	807	8298	4281	175	13	0.001	**
C Loss_700_	µmol m^−2^ s^−1^	−1260	16 737	7105	253.3	13	0.003	**
**Steady‐state traits**								
*A*	µmol m^−2^ s^−1^	19.5	35.1	26.4	59.1	13	0.035	*
*g*_s_	mol m^−2^ s^−1^	0.26	1.45	0.72	166.1	13	0.161	
*C*_i_	µmol mol^−1^	240.7	328	289	30.2	13	0.191	
*E*	mmol m^−2^ s^−1^	4.2	12.9	8.5	102.3	13	0.72	
iWUE	µmol CO_2_ mol^−1^ H_2_O	20.1	76.3	40.3	139.3	13	0.174	

Traits are as follows: ${\bar{A}}_{300}$ and ${\bar{A}}_{700}$, average *A* during the first 300 s and 700 s of induction, respectively; *g*_s avg_, average stomatal conductance during the first 300 s of induction; iWUE_avg_, average intrinsic water‐use efficiency ($\text{iWUE}={\bar{A}}_{300}/g_{\text{s avg}}$); *C*_i avg_, average intercellular [CO_2_] during the first 300 s; *A*_300_, *A* at 300 s into induction; *A*_Max_, maximum rate of CO_2_ uptake across the entire induction period; IT_50_ and IT_90_, time that *A* reached 50% and 90%, respectively, of *A*_300_; IT_90_ *− *IT_50_, the difference between IT_90_ and IT_50_; ${\text{C Loss}}_{300}=\left(A-{\bar{A}}_{300}\right)\times 300$, the difference between the total uptake that would have occurred over the first 300 s if *A* had risen instantaneously to *A*_300_ less the integral of the actual *A* over the first 300 s; *C* Loss_700_ = ${\text{C Loss}}_{700}=\left(A-{\bar{A}}_{700}\right)\times 700$; *A*_sat_, light‐saturated leaf CO_2_ uptake; *g*_s_, stomatal conductance; *C*_i_, intercellular [CO_2_] at steady state; intrinsic water‐use efficiency (iWUE = *A*/*g*_s_); PGV = (Min. – Max.)/Mean. *, *P* < 0.05; **, *P* < 0.01; ***, *P* < 0.001. df, degrees of freedom.

Seeds were maintained at 50°C for 1 wk to break dormancy and then sown into soil from the IRRI Upland Farm in small pots (4.5 cm diameter × 12 cm) and fertilized using 0.4 g l^−1^ of Osmocote Plus 15‐9‐12 (The Scotts Company Ltd, Thorne, UK). Seedlings were transferred to larger pots (21.5 cm diameter × 21.5 cm, 6 l) after the emergence of the second leaf. These were then placed in a screen house, a type of glasshouse with a glass roof but screen‐meshed walls, with no additional lighting or temperature control at IRRI during the Philippines dry season from March to May 2017. Each pot was kept flooded using a drip irrigation system to mimic paddy conditions.

### Gas‐exchange measurements

#### Photosynthetic measurements

Rice plants were dark adapted overnight. The youngest fully expanded leaf, judged by ligule emergence, was placed in the cuvette of an open gas‐exchange system (LI‐6400XT; Li‐Cor, Lincoln NE, USA). Light was provided by an integrated LED head (2 × 3 LED, LI‐6400‐02B). Within the cuvette, air temperature was 28°C, flow rate was 400 µmol s^−1^_,_ [CO_2_] was maintained at 400 µmol mol^−1^, and water vapour pressure deficit at 1–1.6 kPa. For steady‐state measurements, leaves were allowed to reach constant rates of CO_2_ uptake *A* and stomatal conductance *g*_s_ at 1700 µmol m^−2^ s^−1^ photosynthetic photon flux density (PPFD). For induction, leaves were first allowed to reach a steady state in low light of 50 µmol m^−2^ s^−1^ PPFD (‘shade’) for 300 s followed by 720 s at 1700 µmol m^−2^ s^−1^ PPFD (‘sun’). Gas‐exchange measures were logged every 10 s for the duration of the experiment. Measurements were repeated for all 14 accessions (*n* = 4 plants) in the span of 2 d to minimize any time‐dependent effects. Plants were selected at random and measured from 08:00 h to 12:00 h, to avoid confounding accessions with time of day and to minimize any diurnal influences. *A*, *g*_s_, intercellular CO_2_ concentration *C*_i_, transpiration *E*, and intrinsic water‐use efficiency iWUE were calculated following the equations of von Caemmerer and Farquhar (1981). The first 300 s of the induction period were selected to allow a uniform basis for comparing accessions for CO_2_ uptake and speed of induction. This also represents the period in which most change occurs. However, steady state can take many more minutes to attain, albeit with a small remaining change.

The three accessions that showed the most contrasting induction responses, AUS 278, NCS 711 A and IR64‐21, were selected for further analysis of limitations to both induction and steady‐state photosynthesis. Determination of the response of *A*/*C*_i_ followed our previously described protocol (Long & Bernacchi, 2003). Induction was measured following the protocol already described for induction, but at a cuvette [CO_2_] of either 100, 200, 300, 400, 600 or 800 µmol mol^−1^ through the induction. The order of cuvette [CO_2_] treatments for each individual leaf was randomized to avoid confounding [CO_2_] with time. Leaves were dark adapted for a minimum of 1 h between measurements at the different [CO_2_]. To determine limitations through induction, *A* was plotted against *C*_i_ for different time points, following the procedure of Soleh *et al. *(2016). This allowed determination of whether, at any given time point in induction, photosynthesis within the mesophyll was limited by the rate of RuBP regeneration *J*_max_, apparent RuBP carboxylase/oxygenase (Rubisco) activity *V*_c,max_, and to quantitatively partition nonstomatal and stomatal limitations at each time point during induction, following the methods of Bernacchi *et al.* (2003) and Kaiser *et al.* (2017), as outlined in the following.

#### Calculations

Nonstomatal and stomatal limitations were calculated using the following equations, where the subscript ‘time’ refers to the minimum limitation at a given point in time during induction and the subscript ‘final’ refers to the minimum limitation at the final time point during induction. These equations were adapted from those of Kaiser *et al*. (2017). In the absence of diffusional limitation, the CO_2_ uptake rate at a given point in induction would be $A_{C_{a}}^{*}$:

(Eqn 1) $$A_{C_{a}}^{*}=A\times \frac{\min{\left\{A_{c}\left(C_{a}\right),A_{j}\left(C_{a}\right),A_{t}\left(C_{a}\right)\right\}}_{\text{time}}}{\min{\left\{A_{c}\left(C_{i}\right),A_{j}\left(C_{i}\right),A_{t}\left(C_{i}\right)\right\}}_{\text{final}}}$$

(Eqn 2) $$A_{C_{i}}^{*}=A\times \frac{\min{\left\{A_{c}\left(C_{i}\right),A_{j}\left(C_{i}\right),A_{t}\left(C_{i}\right)\right\}}_{\text{time}}}{\min{\left\{A_{c}\left(C_{i}\right),A_{j}\left(C_{i}\right),A_{t}\left(C_{i}\right)\right\}}_{\text{final}}}$$

(*A*, the CO_2_ uptake rate at any given point in the induction; *A*_c_, *A*_j_ and *A*_t_ are the rates that can be supported at that time point and a given [CO_2_] by Rubisco activity, RuBP regeneration, and triose‐phosphate utilization, respectively; *C*_a_, the chamber [CO_2_]; *C*_i_, intercellular [CO_2_]).

Nonstomatal limitation *L*_NS_ and stomatal limitation *L*_S_ were then calculated:

(Eqn 3) $$L_{\text{Nonstomatal}}=\frac{A_{f}-A_{C_{i}}^{*}}{A_{f}-A_{i}}\times 100$$

(Eqn 4) $$L_{\text{Stomatal}}=\frac{A_{C_{a}}^{*}-A}{A_{f}-A_{i}}\times 100$$

(*A*_f_, equal to the final or steady‐state CO_2_ uptake rate at the end of induction; *A*_i_, the value prior to induction during shade). All calculated limitations are relative to the final value of the induction. Since limitations are based on *C*_i_, and not chloroplast [CO_2_], mesophyll conductance will be included in the nonstomatal limitations.

C Loss*_t_*, the integrated amount of CO_2_ uptake lost due to the lower rates through induction compared with steady state, was calculated thus:

(Eqn 5) $${\text{CLoss}}_{t}=\left(A-{\bar{A}}_{t}\right)\times t$$

(*A*, the steady‐state rate of uptake; ${\bar{A}}_{t}$, the average rate across the measured time period from the start of the induction *t*, either 300 s or 700 s).

Percentage genetic variation (PGV) was calculated thus:

(Eqn 6) $$\text{PGV}=\frac{X_{\max}-X_{\min}}{\bar{X}}\times 100$$

(*X*_max_, *X*_min_ and $\bar{X}$, the maximum, minimum and mean values, respectively, of each trait across the 14 accessions).

### Statistical analyses

All statistical analyses and model‐fitting used R (v.3.5.2). Normal distribution and homogeneity of variances were tested by the Shapiro–Wilk test and Brown–Forsythe test, respectively. For data conforming to both assumptions, ANOVA was performed followed by Tukey's mean discrimination analysis, using the R package agricolae. Correlations between measured parameters were assessed using the Pearson correlation analysis (R packages corrplot and hmisc). Accession means were used for the Pearson correlation analysis.

## Results

### Photosynthetic induction responses vary significantly between 14 rice accessions

**Figure 1:**
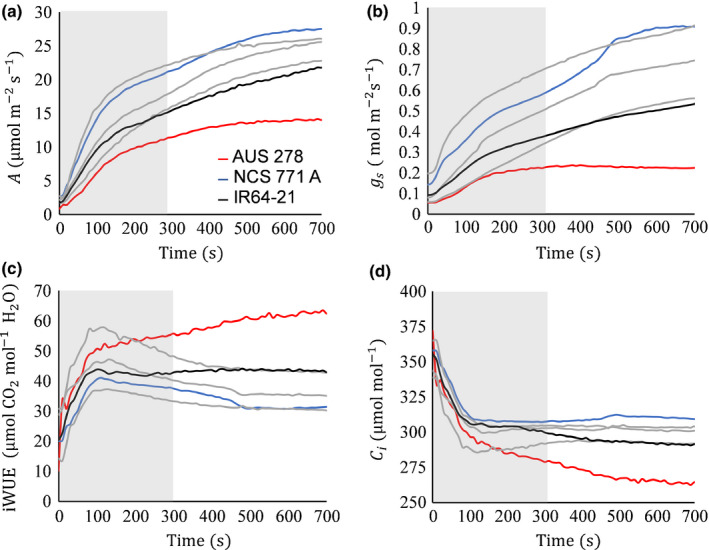
(a) Net leaf CO_2_ uptake *A*, (b) stomatal conductance *g*_s_, (c) intrinsic water use efficiency iWUE = *A*/*g*_s_, and (d) intercellular [CO_2_] *C*_i_ with time *t* of induction on transfer at 0 s from low to high light (50–1700 µmol m^−2^ s^−1^) in rice (*Oryza sativa*). The ‘induction period’, characterized by a steep increase in *A*, is demarked with the grey box and lasted up to 300 s. The three accessions that were selected for further study of induction at varied [CO_2_] were AUS 278 (red), NCS 771 A (blue) and IR64‐21 (black). The other three accessions in grey are Du Gen Chuan, K2 C45 and Malogbana. For ease of visualization, only six accessions are included, but the data for all 14 accessions are available in Table 1 and Fig. 2.

Induction of CO_2_ uptake and stomatal conductance showed biphasic responses to a change from 50 to 1700 µmol m^−2^ s^−1^ PPFD. During the first 120 s of induction, *A* and *g*_s_ both increased rapidly, followed by a more gradual increase (Fig. 1). At steady state, the only trait that varied significantly between accessions was *A* (*P* = 0.035) (Table 1), whereas almost all traits showed significant variation under the nonsteady‐state conditions of induction (Fig. 1; Table 1). Nonsteady‐state measurements showed *c.* 20–40% greater variation between accessions relative to the equivalent trait at steady state (Table 1).

**Figure 2:**
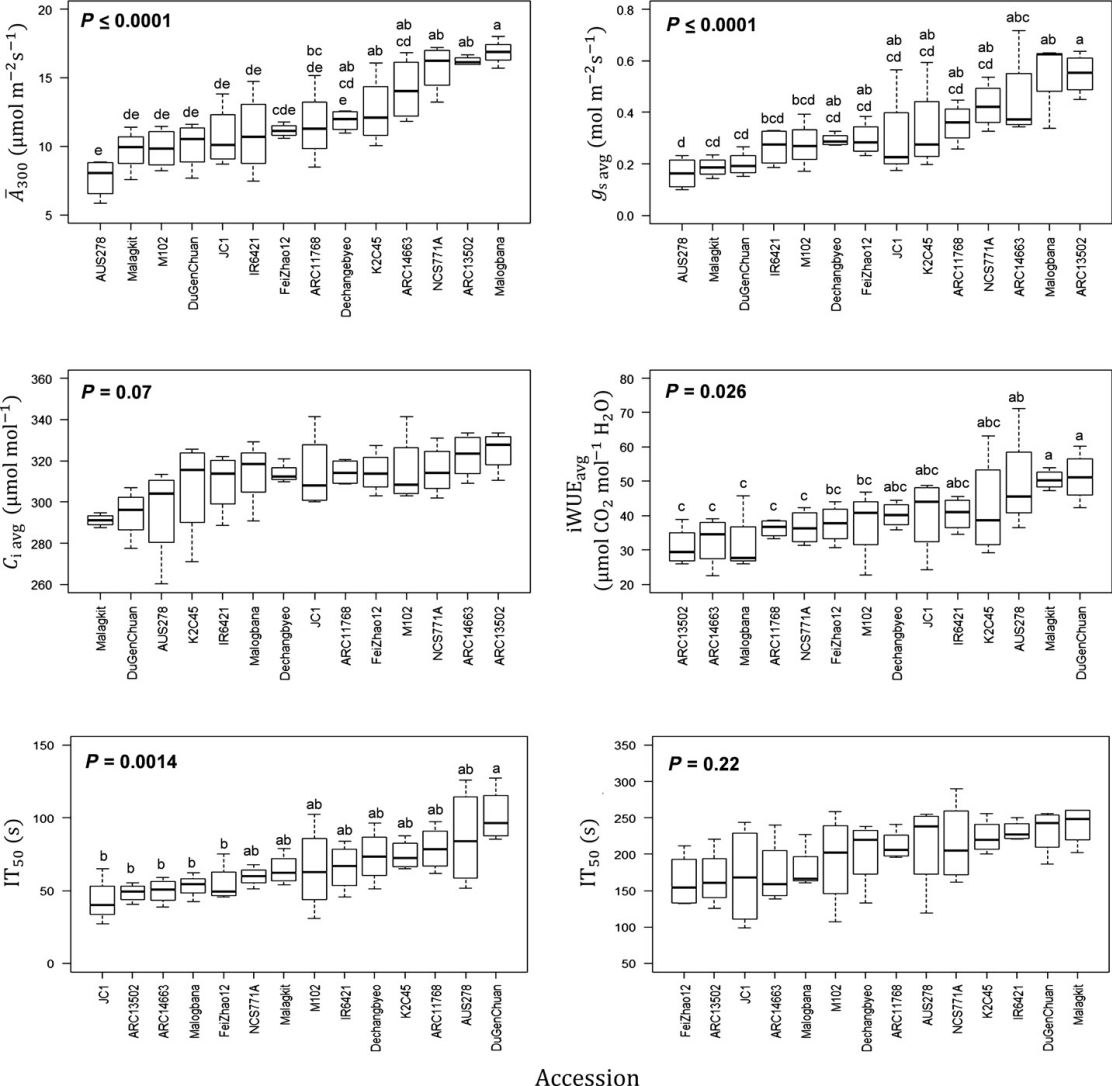
Mean and variation for all rice (*Oryza sativa*) accessions for average leaf CO_2_ uptake during the first 300 s of induction (${\bar{A}}_{300}$), average A stomatal conductance during the first 300 s of induction (*g*_s avg_), average intrinsic water‐use efficiency (${\text{iWUE}}_{\text{avg}}={\bar{A}}_{300}/g_{\text{s avg}}$), average intercellular CO_2_ concentration during the first 300 s (*C*_i avg_), and time that *A* reached 50% and 90% of *A*_300_ (IT_50_ and IT_90_, respectively). The accessions are ranked by increasing mean for each parameter. Different letters represent statistically significant differences (*P* < 0.05) between different accessions.

Averaged across the induction period, CO_2_ uptake ${\bar{A}}_{300}$, stomatal conductance *g*_s avg_, intrinsic water‐use efficiency during induction iWUE_avg_, maximum CO_2_ uptake during induction *A*_Max_, and the time to 50% induction in seconds IT_50_ all varied significantly between accessions (Table 1). The substantial range of variation was evident in PGV: 102% for ${\bar{A}}_{300}$, 190% for *g*_s avg_, 121% for iWUE_avg_, and 152% for IT_50_ (Table 1). There were no significant differences in *C*_i avg_, the average *C*_i_ over the induction (Table 1). The highest and lowest performing accession means differed 109% with respect to ${\bar{A}}_{300}$ (*P* ≤ 0.0001) (Figs 1, 2). These differences between accessions were independent of subpopulation, geographic region, or canopy structure (Figs 2, S1, S2; Table S1). The accession mean that had the highest ${\bar{A}}_{300}$ was Malogbana (16.8 µmol m^−2^ s^−1^), an admixed accession from Cote d'Ivoire, whereas the lowest was AUS 278 (7.6 µmol m^−2^ s^−1^), an aus accession from Bangladesh (Fig. 2; Table S1). There was a 65% difference in iWUE_avg_, between accession means over the first 300 s of induction (50.2–30.9 µmol CO_2_ mol^−1^ H_2_O; Fig. 2). Additionally, there was a significant 117% difference in IT_50_ between accessions (Fig. 2). The accession showing the slowest induction, as determined by IT_50_, was Du Gen Chuan, a Chinese *indica* accession (101 s), whereas the fastest was JC1, a Chinese aromatic accession (43 s; Fig. 2; Table S1). Despite significant differences in IT_50_, the time to 90% induction IT_90_ did not vary significantly between accessions, indicating that most of the variation occurs in the early phase of induction (Fig. 2; Table 1). Loss of potential C fixation due to the lag that occurs in photosynthesis through the first 300 s of induction (C Loss_300_) and 700 s (C Loss_700_) varied significantly between accessions (Table 1).

### No significant relationship between steady‐state and induction measures of photosynthesis

**Figure 3:**
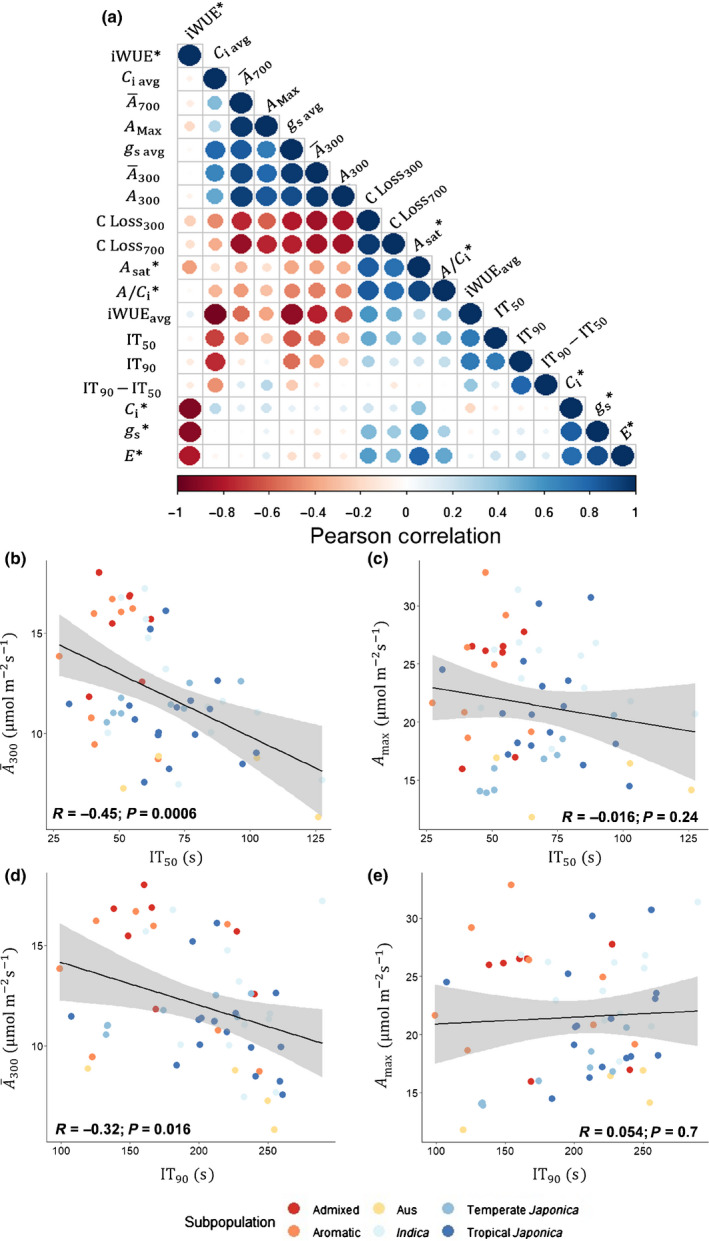
(a) Pearson correlation *R* ^2^ of all measured dynamic and steady‐state (*) photosynthetic traits measured in rice (*Oryza sativa*). Negative correlations (red) and positive (blue). Traits are as follows: ${\bar{A}}_{300}$ and ${\bar{A}}_{700}$, average *A* during the first 300 s an 700 s of induction, respectively; *A*_sat_, light‐saturated leaf CO_2_ uptake; *g*_s_, stomatal conductance; *C*_i_, intercellular [CO_2_] at steady state; iWUE, intrinsic water‐use efficiency (iWUE* = A*/*g*_s_); *g*_s avg_, average stomatal conductance during the first 300 s of induction; iWUE_avg_, average intrinsic water‐use efficiency ($\text{iWUE}={\bar{A}}_{300}/g_{\text{s avg}}$); *C*_i avg_, average intercellular [CO_2_] during the first 300 s; *A*_300_, *A* at 300 s into induction; *A*_Max_, maximum rate of CO_2_ uptake across the entire induction period; IT_50_ and IT_90_, time that *A* reached 50% and 90%, respectively, of *A*_300_; IT_90_ *− *IT_50_, the difference between IT_90_ and IT_50_; ${\text{C Loss}}_{300}=\left(A-{\bar{A}}_{300}\right)\times 300$, the difference between the total uptake that would have occurred over the first 300 , if *A* had risen instantaneously to *A*_300_ less the integral of the actual *A* over the first 300 s; ${\text{C Loss}}_{700}=\left(A-{\bar{A}}_{700}\right)\times 700$. (b–e) Individual measures, regression line, correlation coefficient *R* ^2^, confidence interval (95%), and *P*‐value for (b) ${\bar{A}}_{300}$ vs IT_50_, (c) ${\bar{A}}_{300}$ vs IT_90_, (d)* A*_Max_ vs IT_50_, and (e) *A*_Max_ vs IT_90_.

There was no significant correlation between ${\bar{A}}_{300}$ or the speed of induction and *A* at steady state and, indeed, no significant correlation between any induction trait and its steady‐state equivalent (Fig. 3). Correlations were found between different measures made within light induction and within steady state, but not between. Among induction traits, significant positive correlations were found between ${\bar{A}}_{300}$ and *g*_s avg_, ${\bar{A}}_{300}$ and $A_{\text{Max}}$, and ${\bar{A}}_{300}$ and ${\bar{A}}_{660}$ (Fig. 3). Significant negative correlations were found between ${\bar{A}}_{300}$ and iWUE_avg_, iWUE_avg_ and *g*_s avg_, and ${\bar{A}}_{300}$ and ${\text{CLoss}}_{300}$ (Fig. 3). Interestingly, a significant negative correlation was found between ${\bar{A}}_{300}$ and speed of induction, with plants that had a lower IT_50_ and IT_90_ assimilating more CO_2_ (Fig. 3b,d). Additionally, faster stomatal opening and greater *g*_s_ at the beginning of induction were significantly correlated with greater ${\bar{A}}_{300}$ and quicker IT_50_ and IT_90_ (Figs S3, S4). Furthermore, plants responded consistently for *g*_s_ throughout induction, as indicated by a strong correlation (*P* = 0.0085); that is, a high *g*_s_ early in induction was consistent with a high *g*_s_ at the end of induction (Fig. S4). For measures made at steady‐state, positive correlations were predictably found between *g*_s_ and *C*_i_ and between *g*_s_ and *A*, and negative correlations between iWUE and *g*_s_ and between iWUE and *C*_i_ (Fig. 3).

### Rice photosynthetic induction is mainly limited by biochemistry

**Figure 4:**
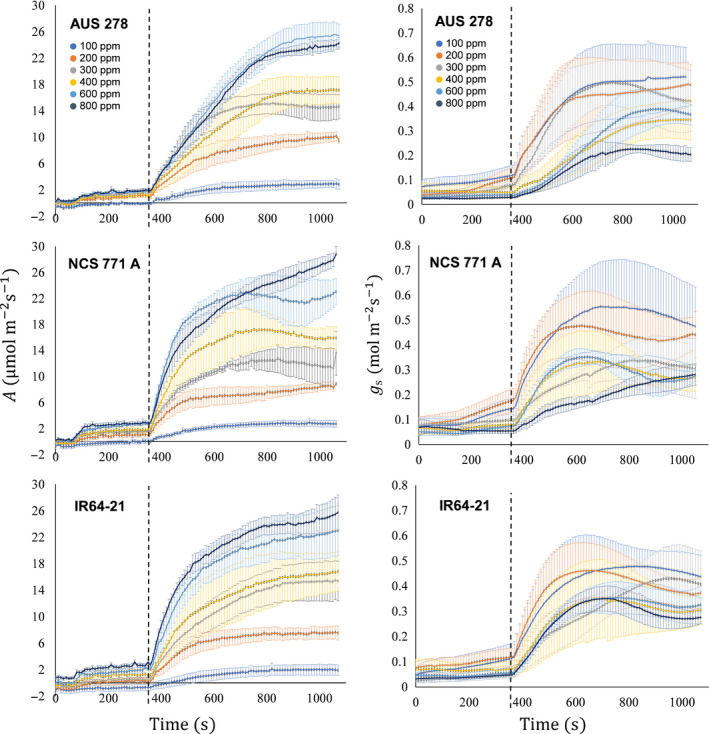
Induction of leaf CO_2_ uptake *A* and stomatal conductance *g*_s_ at [CO_2_] = 100, 200, 300, 400, 600 and 800 µmol mol^−1^ for three rice (*Oryza sativa*) cultivars. Each point is the mean (± SE) of four plants. The dashed vertical line at 360 s represents the point of transition from low to high photosynthetic photon flux density (50–1700 µmol m^−2^ s^−1^); that is, the start of induction.

**Figure 5:**
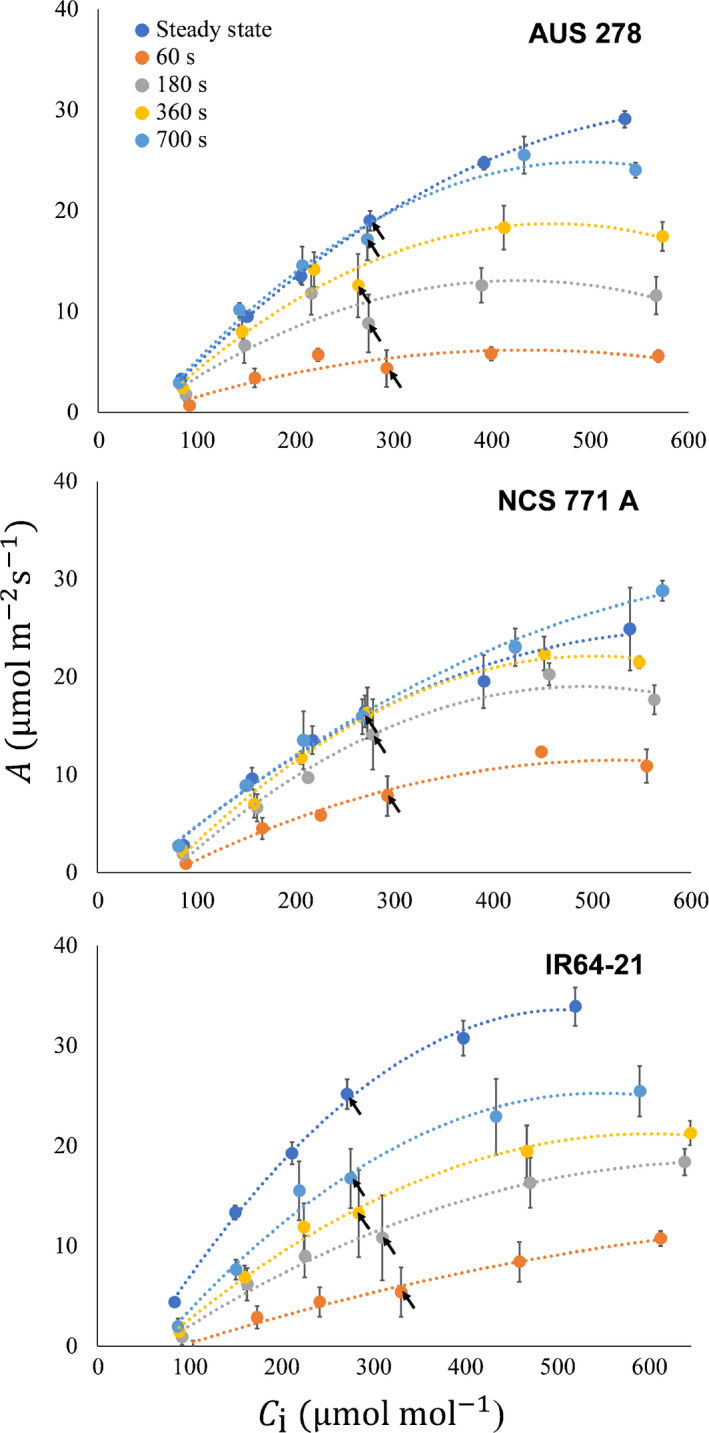
The responses of leaf CO_2_ uptake *A* to intercellular [CO_2_] *C*_i_ at steady state (dark blue), and at 60 s (orange), 180 s (grey), 360 s (yellow), and 700 s (light blue) from the start of induction and replotted from Fig. 4. The operating point of each curve at 400 µmol mol^−1^ atmospheric [CO_2_] *C*_a_ is indicated with a black arrow. Each point is the mean (± SE) of four plants of each rice (*Oryza sativa*) accession.

Three accessions were selected, based upon the variation in their ${\bar{A}}_{300}$: NCS 771 A, IR64‐21 and AUS 278; 15.7 m^−2^ s^−1^, 10.9 m^−2^ s^−1^ and 7.7 m^−2^ s^−1^, respectively. Induction was measured on the three selected accessions at six [CO_2_] (Fig. 4). Dynamic *A*/*C*_i_ curves were constructed at four time points: 60, 180, 360 and 700 s after the beginning of induction (Fig. 5). The temporal changes in the dynamic *A*/*C*_i_ curves were highly dependent upon the accession (Fig. 5). When compared, dynamic curves for NCS 771 A strongly approximated the steady‐state *A*/*C*_i_ curves at both 360 s and 700 s. Dynamic *A*/*C_i_* curves in AUS 278 were only similar to the steady‐state curve at 700 s, whereas *A* values for IR64‐21 were lower than at steady state at all *C*_i_s, even at 700 s (Fig. 5).

**Figure 6:**
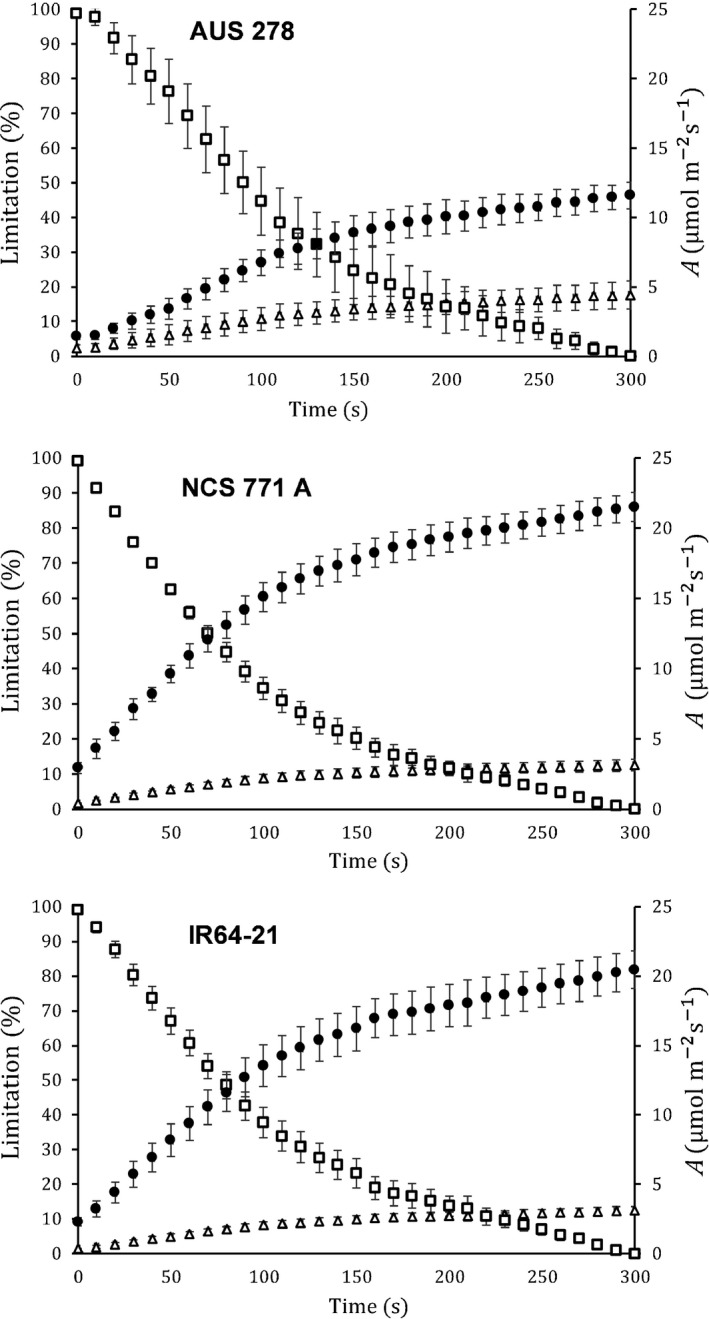
Nonstomatal (open squares) and stomatal (closed triangles) limitations with leaf CO_2_ uptake *A* (closed circles) vs time over the first 300 s of induction for the three selected rice (*Oryza sativa*) accessions. Nonstomatal and stomatal limitations during induction were calculated relative to the near‐steady‐state value obtained at 300 s. Each point represents the mean (± SE) of four individual plants measured at ambient [CO_2_].

When stomatal and nonstomatal limitations were calculated relative to those at steady state for all three accessions, nonstomatal limitation *L*_NS_ accounted for almost 100% initially, declining to 30% at 120 s and then to no more limiting than at steady state (Fig. 6). In contrast, stomatal limitation *L*_S_ gradually increased from *c.* 2% to *c.* 10–15% over the first 300 s (Fig. 6). Similar patterns were seen in IR64‐21, AUS 278 and NCS 771 A, although *L*_NS_ decreased more rapidly in NCS 771 A. Consistent with strong *L*_NS_, *C*_i avg_ was higher during induction than *C*_i_ at steady state (Table 1). This is reflected in the dynamic *A*/*C*_i_ responses, where *C*_i_/*C*_a_ was higher than at steady state at all [CO_2_], again indicating that stomatal conductance was less of a limitation during induction than at steady state (Fig. 6). Since estimates of *L*_NS_ and *L*_S_ were based on *C*_i_, any limitation due to mesophyll conductance is included in *L*_NS_ *.*

**Figure 7:**
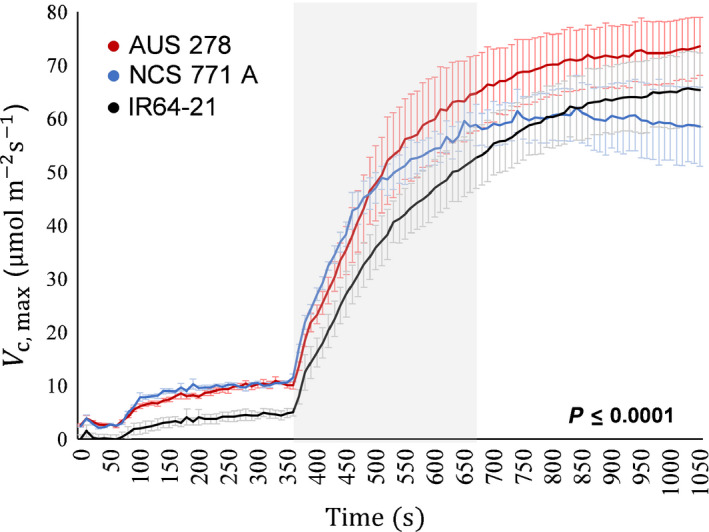
Apparent maximum rate of carboxylation *V*_c,max_ with time through induction calculated from the response of leaf CO_2_ uptake *A* to intercellular [CO_2_] *C*_i_ derived from inductions made at different [CO_2_], as in Fig. 4. Induction began at 360 s. Significance of difference between the three rice (*Oryza sativa*) accessions is based on a repeated measures (time) one‐way ANOVA. Each point represents the mean (± SE) of four individual plants.

The dynamic *A*/*C*_i_ curves suggested that photosynthetic induction was strongly limited by Rubisco (Fig. 5). Fitting *V*_c,max_ to the *A*/*C*_i_ responses showed its sharp increase during the first 300 s of induction, followed by a more gradual increase to steady‐state values (Fig. 7). *V*_c,max_ during induction differed significantly between the three accessions (Fig. 7).

## Discussion

### Possibilities for exploiting natural variation in the photosynthetic induction response of rice

Natural genetic variation is one of the key drivers of evolution and is essential for plant improvement through breeding. Although breeding selection for higher productivity has resulted in only very small increases in leaf photosynthetic capacity, recent studies have suggested variation within crop diversity panels could be exploited for selection based on direct measurement of photosynthesis (Flood *et al.*, 2011; Lawson *et al.*, 2012; Driever *et al.*, 2014; Gu *et al.*, 2014; Koester *et al.*, 2016). However, these studies have only examined steady‐state photosynthesis. Given that photosynthesis is probably never at steady state in crop fields, the speed of adjustment to dynamic lighting may be as or more important. Recent studies have suggested that speeding up the rate of adjustment to sunlight‐to‐shade and shade‐to‐sunlight transitions could each increase canopy CO_2_ uptake in the field, each by *c.* 20% (Kromdijk *et al.*, 2016; Taylor & Long, 2017).

Here, gas‐exchange methods were used to determine the potential extent of diversity and identify useful parameters. Though only a few genotypes could be examined by these methods, they represent a broad cross‐section of rice germplasm (Table S1). Significant natural variation was found between accessions for the nonsteady‐state measures ${\bar{A}}_{300}$, IT_50_ and iWUE_avg_, indicating variation in rice that could be exploited to improve photosynthesis in dynamic conditions. Improving the speed of photosynthetic induction would be particularly desirable as it would allow the plant to respond more rapidly to fluctuations in its light environment, capitalizing on available photosynthetically active radiation (Taylor & Long, 2017). These improvements in photosynthetic induction could then translate to increases in plant productivity, especially if combined with increased overall photosynthetic capacity (Taylor & Long, 2017). Indeed, IT_50_ was strongly correlated with ${\bar{A}}_{300}$, suggesting that both parameters could be improved simultaneously (Fig. 3). However, as in steady‐state conditions, trade‐offs exist between photosynthetic performance and water‐use efficiency, which need to be taken into consideration. Undoubtedly, breeding for increased photosynthetic efficiency in fluctuating light would require screening and analysis of more genotypes than examined here. Modulated Chl fluorescence imaging would be an effective method to screen large diversity collections and the progeny of any crosses. The current study has shown biochemical limitations, rather than stomatal limitations, as the major factor influencing CO_2_ uptake during the first few minutes of a shade to sun transition in rice. Biochemical limitation could be screened through fluorescence imaging by monitoring the increase in efficiency in photosystem II through induction of leaf disks in 96‐well plates, for example. This would allow the screening of several hundred genotypes in a day (Murchie & Lawson, 2013).

There was no obvious association of induction parameters with geographic region or genetic grouping, suggesting wide crosses would not be needed to obtain variation in these parameters for selection. Here, we also found that crop canopy (Fig. S1) did not correlate with photosynthetic performance during induction (Fig. S2). Despite their contrasting photosynthetic induction performances, both NCS 771 A and AUS 278 had drooping canopies, whereas IR64‐21 had an erect canopy structure (Fig. S1). Additionally, it was found that IR64, an elite high‐yielding variety released in 1985, was significantly outperformed by several accessions for both dynamic and steady‐state photosynthetic traits (Fig. 1). At the height of its popularity, IR64 was farmed on over 10 × 10^6^ ha world‐wide while accounting for 70% of rice area planted in Indonesia and 10% of breeder seed produced in India (Mackill & Khush, 2018). IR64 is still widely cultivated across much of tropical Southeast and South Asia, although it has been replaced by newer varieties, many of which are its progeny or relatives (Mackill & Khush, 2018). IR64 was bred by initially selecting for semi‐dwarf morphology, increasing harvest index but not photosynthetic performance (Mackill & Khush, 2018). This study underscores the importance of screening landraces and accessions that are not widely cultivated, as they may perform better than elite varieties, with respect to photosynthesis in fluctuating light, and act as a source for plant improvement. There is also untapped potential to improve photosynthetic induction by screening wild rice species and the progenitors of rice (*Oryza rufipogon* and *Oryza nivara*) which can exhibit greater rates for leaf CO_2_ uptake when compared to cultivated *Oryza sativa* accessions (Zhao *et al.*, 2008).

### How [CO_2_] influences the response of photosynthetic induction

The response of photosynthetic induction to elevated [CO_2_] is an important consideration given the current rapid rise in global atmospheric [CO_2_] (IPCC, 2018). Previous in‐field studies that have focused almost exclusively on steady‐state photosynthesis show a large increase in CO_2_ uptake of 25–50% almost in proportion to the increase in [CO_2_]. However, yield increases measured under nonsteady‐state open‐air field elevation of [CO_2_] in rice are considerably less than the increases predicted from controlled environment studies (Long *et al.*, 2006; Ainsworth, 2008; Cai *et al.*, 2015). Here, we found that rice saw increased leaf CO_2_ uptake in response to elevated [CO_2_]. However, ${\bar{A}}_{300}$ increased on average by 43% between 400 and 800 µmol mol^−1^, whereas steady‐state *A* increased by 53% (Fig. 4), showing that the increases are smaller in fluctuating light than at steady state. This could be part of the explanation as to why productivity gains in field conditions are smaller than those observed under elevated [CO_2_] in controlled environments.

### Photosynthetic induction in rice is limited mostly by biochemistry

Here, *C*_i_ was consistently higher during induction despite having lower *A*, indicating a biochemical rather than stomatal limitation to photosynthesis (Table 1). *L*_NS_ was found to be more limiting than *L*_S_, except in the final phase of induction when *L*_S_ became more prominent (Fig. 5). Analysis of the *A*/*C*_i_ response indicated *V*_c,max_ to be limiting throughout induction in all three accessions (Fig. 5), similar to previous results across a wide range of genotypes in soybean (Soleh *et al.*, 2017). As noted in the Results section and as in the Soleh *et al. *(2017) study, our methods do not separate limitations due to mesophyll conductance from biochemical limitations. Changes in mesophyll conductance have been suggested to be far more rapid than induction of carboxylation, but this clearly requires further investigation (Deans *et al.*, 2019).

Limitations to photosynthetic induction appear to be species dependent. Soy and wheat are primarily limited by their biochemistry, whereas cassava and some tropical tree species are more heavily limited by stomata (Tinoco‐Ojanguren & Pearcy, 1993; Valladares *et al.*, 1997; Soleh *et al.*, 2016; Taylor & Long, 2017; de Souza, *et al.*, 2019). Species that have a weaker coupling between *A* and *g*_s_ responses in dynamic light conditions have greater limitation from stomata and exhibit a stronger lag in *g*_s_ in reaching steady state (McAusland *et al.*, 2016). In the present study, stomata only accounted for *c.* 10% of limitation to *A* during photosynthetic induction (Fig. 5), similar to that found for rice by McAusland *et al.*, 2016. The dumb‐bell‐shaped guard cells in rice may contribute to a faster stomatal response as they require fewer solutes and less water for a change in aperture, allowing them to respond more quickly than elliptical‐shaped guard cells (McAusland *et al.*, 2016). Additionally, plants with smaller stomata, such as rice, are known to respond more quickly to environmental stimuli, which may reduce limitation by stomata in dynamic conditions (Ohsumi *et al.*, 2007; Drake *et al.*, 2013; Raven, 2014). It is possible that *L*_S_ may not play such a strong role in rice induction. Its wild ancestors were emergent aquatic plants, and most breeding programmes that target the improvement of lowland rice utilize flooded paddies, where water is not limiting. After millennia of cultivating rice in conditions with plentiful water, it is likely that rice is less conservative in its water usage, explaining the lower *L*_S_ (Nay‐Htoon *et al.*, 2018).

### The lack of correlation between dynamic and steady‐state photosynthesis in rice

Here, no significant relationship was found between steady‐state and the equivalent nonsteady‐state trait during induction (Fig. 2), paralleling prior work with soybean (Soleh *et al.*, 2016). Accessions that had high CO_2_ uptake during induction or a speedier induction rate did not necessarily have higher CO_2_ uptake in steady‐state measurements (Fig. 2). For example, rates of induction that appear dominated by Rubisco activation are not correlated with steady‐state *V*_c,max_, suggesting a lack of correlation between Rca activity and Rubisco activity at steady state.

The lack of correlation between the steady‐state photosynthetic phenotype and induction phenotypes challenges the way that photosynthesis has conventionally been measured and understood for selection in the field environment. In the absence of significant correlations between dynamic and steady‐state photosynthetic phenotypes, therefore, criteria used in the selection of photosynthetic efficiency in crop improvement of productivity need to be rethought. The results here indicate that consideration of steady‐state criteria alone has likely failed to account for the larger part of phenotypic diversity in crop germplasm with respect to photosynthesis. Variation across the germplasm examined in photosynthetic traits during induction was 40% greater than in their steady‐state counterparts (Table 1). Though improving photosynthesis remains a major opportunity for improving genetic yield potential (Evans, 1997; Zhu *et al.*, 2008, 2010; Long *et al.*, 2015), it appears that more focus on nonsteady‐state traits is needed.

## Author contributions

LGA‐S and SPL planned the research; SPL and WPQ supervised the project. LGA‐S and RC conducted the experimental work, LGA‐S analysed the data, LGA‐S and JK conducted limitations analysis, YW conducted the modelling, and LGA‐S and SPL wrote the manuscript with the input of all the other authors.

## Supporting information

Please note: Wiley Blackwell are not responsible for the content or functionality of any Supporting Information supplied by the authors. Any queries (other than missing material) should be directed to the *New Phytologist* Central Office.

**Fig. S1** 14 selected rice accessions during mid‐tillering to show differences in canopy.
**Fig. S2** Relationship between average CO_2_ uptake during induction (${\bar{A}}_{300}$) and leaf angle, the time to 50% induction (IT_50_) and leaf angle.
**Fig. S3** Correlations between average CO_2_ uptake during induction (${\bar{A}}_{300}$) and time to 50% stomatal opening (*g*_s 50_), time to 90% stomatal opening (*g*_s 90_), time to 50% induction (IT_50_), and time to 90% induction (IT_90_).
**Fig. S4** Correlations between initial and final stomatal conductance during induction and speed of induction.
**Table S1** Description of selected accessions.
**Table S2** SNP sequences for Rubisco activase for the 14 selected accessions.
