# Supplementary material for: Variation in photosynthetic induction between rice accessions and its potential for improving productivity
Source: New Phytol. 2020 Mar 3;227(4):1097–108. doi: 10.1111/nph.16454 (PMC7383871; doi:10.1111/nph.16454)
Supplement: Supplementary file 1 — Fig. S1 14 selected rice accessions during mid‐tillering to show differences in canopy. Fig. S2 Relationship between average CO2 uptake during induction (A¯300) and leaf angle, the time to 50% induction (IT50) and leaf angle. Fig. S3 Correlations between average CO2 uptake during induction (A¯300) and time to 50% stomatal opening (g s 50), time to 90% stomatal opening (g s 90), time to 50% induction (IT50), and time to 90% induction (IT90). Fig. S4 Correlations between initial and final stomatal conductance during induction and speed of induction. Table S1 Description of selected accessions. Table S2 SNP sequences for Rubisco activase for the 14 selected accessions. [file NPH-227-1097-s001.pdf]

New Phytologist Supporting Information Figs S1–S4 and Tables S1 & S2

Article title: Variation in photosynthetic induction between rice accessions and its potential for improving productivity

Authors: Liana G. Acevedo-Siaca, Robert Coe, Yu Wang, Johannes Kromdijk, W. Paul Quick and Stephen P. Long. Article acceptance date: 13 January 2020.

SUPPLEMENTARY FIGURES AND TABLES:

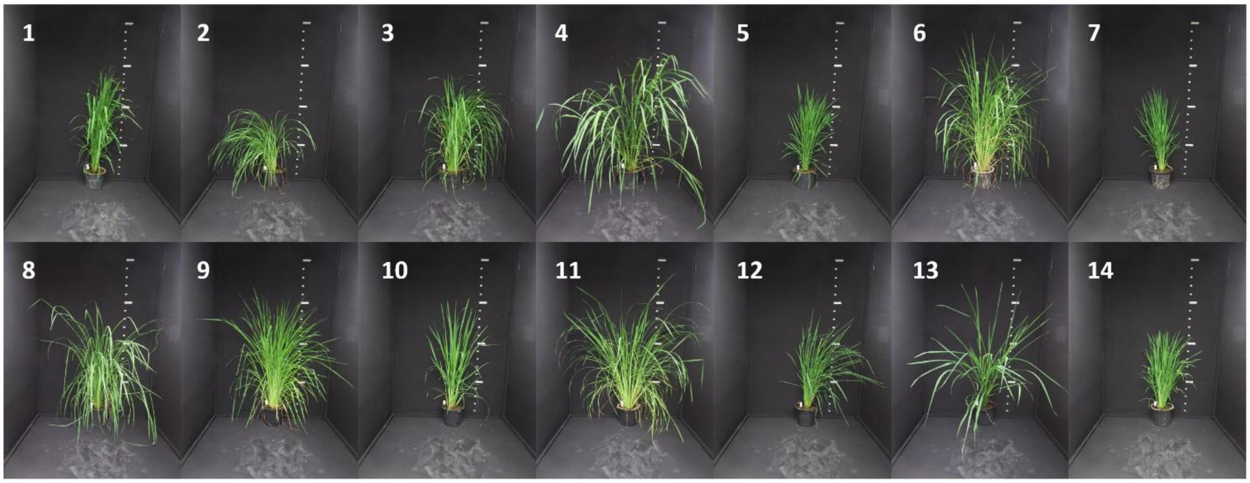

Fig. S1 Selected rice (*Oryza sativa*) accessions are as follow: 1 – ARC 11768, 2 – ARC 13502, 3 – ARC 14663, 4 – AUS 278, 5 – Dechangbyeo, 6 – Du Gen Chuan, 7 – Fei Zhao 12, 8 – JC1, 9 – K2 C45, 10 – Malagkit, 11 – NCS 771A, 12 – M 102, 13 – Malogbana, 14 – IR64-21.

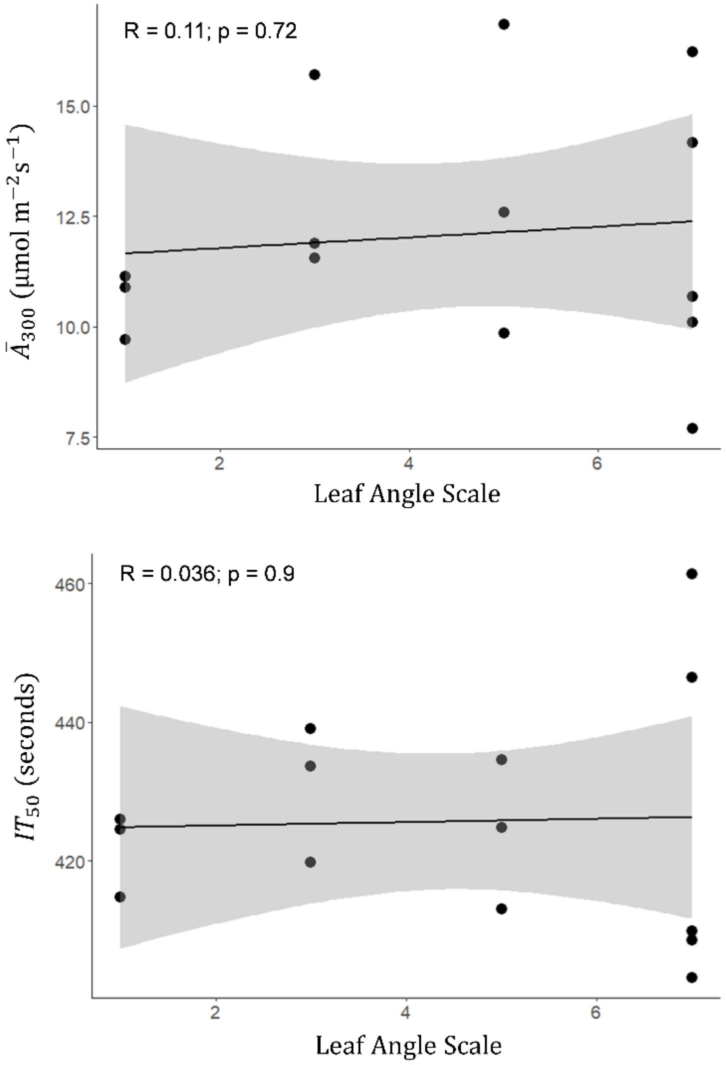

Fig. S2 The correlation between $\bar{A}_{300}$ and Leaf Angle, IT_{50} and Leaf Angle. Rice (*Oryza sativa*) leaf angle can be rated on a scale from 1-9, where 1 is most erect and 9 is most drooping (IRRI Standard Evaluation System (SES) for Rice, 2002). Correlation coefficient (R), p-value, regression line, and 95% confidence intervals are shown for each comparison.

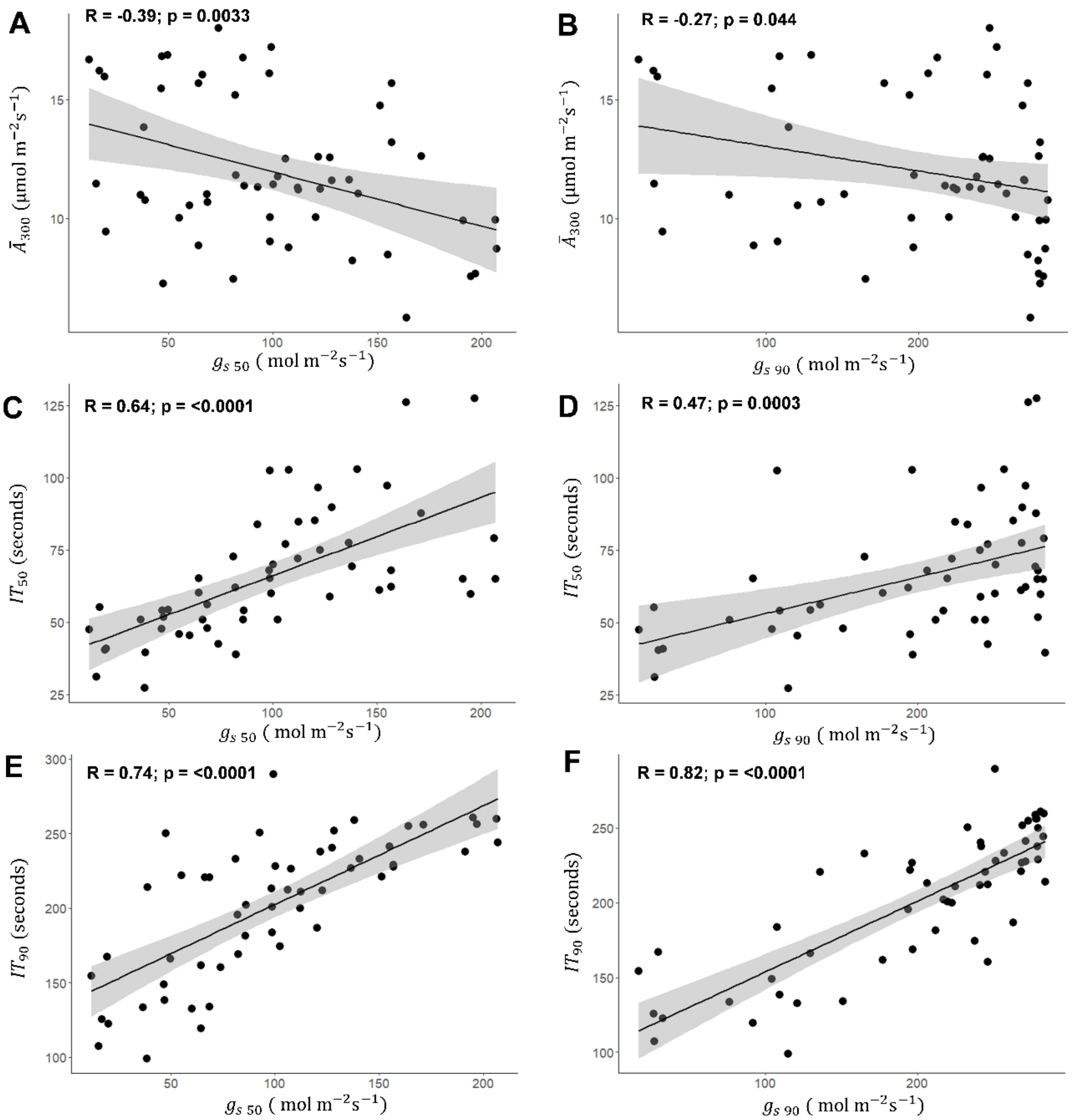

Fig. S3 Correlation between **A.** time to 50% stomatal opening ($g_{s\,50}$) and average CO_2 uptake during induction period ($\bar{A}_{300}$); **B.** time to 90% stomatal opening ($g_{s\,90}$) and $\bar{A}_{300}$; **C.** time to 50% induction (IT_{50}) and $g_{s\,50}$; **D.** IT_{50} and $g_{s\,90}$; **E.** time to 90% induction (IT_{90}) and $g_{s\,50}$**; F.** IT_{90} and $g_{s\,90}$, measured in rice (*Oryza sativa*). Correlation coefficient (R), p-value, regression line, and 95% confidence intervals are shown for each comparison.

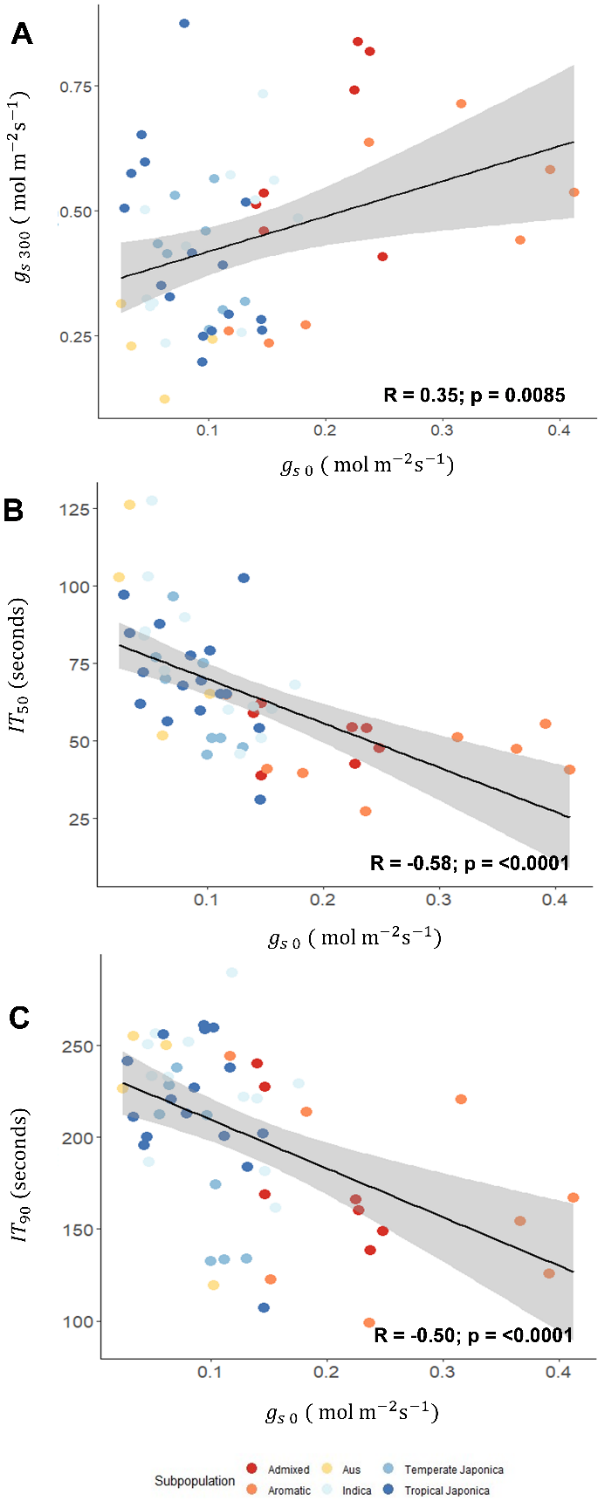

Fig. S4 Correlation between **A.** g_s right before transition from low light to high light (50 to 1700 µmol m^{-2} s^{-1} PPFD) ($g_{s\,0}$) and at 300 s after induction ($g_{s\,300}$); **B.** $g_{s\,0}$ and IT_{50}; **C.** $g_{s\,0}$ and IT_{90}, measured in rice (*Oryza sativa*). Correlation coefficient (R), p-value, regression line, and 95% confidence intervals are shown. Plants are color-coded by subpopulation.

Table S1 *O. sativa* accessions selected from the 3000 Rice Genome Project (3K RGP) used to characterize natural genetic variation for photosynthetic induction in rice. The IRGC number, subpopulation, and country of origin are indicated.

Accession #	Accession Name	IRGC Number	Subpopulation	Geographic Region
**1**	ARC 11768	IRGC 131966	Tropical Japonica	India
**2**	ARC 13502	IRGC 127948	Aromatic	India
**3**	ARC 14663	IRGC 127959	Admixed	India
**4**	AUS 278	IRGC 127182	Aus	Bangladesh
**5**	Dechangbyeo	IRGC 128275	Temperate Japonica	South Korea
**6**	Du Gen Chuan	IRGC 127345	Indica	China
**7**	Fei Zhao 12	IRGC 128291	Temperate Japonica	China
**8**	JC1	IRGC 117494	Aromatic	India
**9**	K2 C45	IRGC 131995	Tropical Japonica	Cote d'Ivoire
**10**	Malagkit	IRGC 127594	Tropical Japonica	Philippines
**11**	NCS 771A	IRGC 127665	Indica	India
**12**	M 102	IRGC 125620	Tropical Japonica	United States
**13**	Malogbana	IRGC 128379	Admixed	Cote d'Ivoire
**14**	IR64-21	IRGC 135929	Indica	Philippines

Table S2 SNP sequences for Rubisco activase (Rca) gene in rice (*Oryza sativa*). Sequences for Rca were acquired through the International Rice Informatics Consortium (IRIC) Rice SNP-seek Database (http://snp-seek.irri.org/_snp.zul) managed by the International Rice Research Institute (IRRI).

			SNP Position					
Accession	Assay ID	IRGC #	28934599	28934614	28935449	28935808	28936124	28936221
JC 1	IRIS 313-8326	IRGC 117494	C	T	T	C	C	G
NCS 771A	IRIS 313-11646	IRGC 127665	C	T	T	C	C	G
IR64	CX230	IRGC 135929	C	T	T	C	C	G
ARC 11768	IRIS 313-10870	IRGC 131966	C	T	T	C	G	G
ARC 13502	IRIS 313-11258	IRGC 127948	C	T	T	C	G	G
Dechangbyeo	IRIS 313-11689	IRGC 128275	C	T	T	C	G	G
Fei Zhao 12	IRIS 313-11651	IRGC 128291	C	T	T	C	G	G
K 2 C 45	IRIS 313-11759	IRGC 131995	C	T	T	C	G	G
Malagkit	IRIS 313-12272	IRGC 127594	C	T	T	C	G	G
M 102	IRIS 313-8502	IRGC 125620	C	T	T	C	G	G
AUS 278	IRIS 313-11052	IRGC 127182	C/T	T	T	C	C	G
ARC 14663	IRIS 313-11270	IRGC 127959	T	T	T	C	C	G
Du Gen Chuan	IRIS 313-11796	IRGC 127345	T	T	T	C	C	G
Malogbana	IRIS 313-10729	IRGC 128379	T	T	T	C	C	G
